# Let's talk about death, dying, and what matters most to you in life: Pretest and piloting of a translated and adapted conversation game

**DOI:** 10.1016/j.pecinn.2025.100400

**Published:** 2025-05-06

**Authors:** Julia Jaschke, Sara Söling, Juliane Köberlein-Neu

**Affiliations:** Center for Health Economics and Health Services Research, Schumpeter School of Business and Economics, University of Wuppertal, Wuppertal, Germany

**Keywords:** Advance care planning, Gamification, Acceptability, Comprehensibility

## Abstract

**Objective:**

This study aimed to pretest and pilot the German version of the *Hello* conversation game (German: *Hey du*) to assess its comprehensibility and acceptability. Like the original, *Hey du* is designed as a low-threshold method to empower people of almost all ages and health conditions to engage in advance care planning (ACP), particularly targeting young/healthy people for whom there are currently few or no ACP programmes.

**Methods:**

The conversation game was systematically translated into German using the TRAPD process and cognitively pretested in 12 interviews. Subsequently, two observational studies were conducted: *Hey du* was used in 1) a nursing school (*n* = 16) and 2) with groups of family and friends (*n* = 50). After completing the game, participants were surveyed regarding the game's acceptability and comprehensibility.

**Results:**

The results of the cognitive pretest and the observational studies show that the conversation game was acceptable and comprehensible in both settings. Most of the participants (>92 %) reported that *Hey du* helped them come to terms with their own wishes and preferences for medical and nursing care and that they felt comfortable playing the game.

**Conclusion:**

*Hey du* has the potential to motivate and empower people to deal with ACP.

**Innovation:**

*Hey du* is the first scientifically supported gamification approach in Germany to introduce ACP to people for whom no systematic ACP programs exist. The game provides a safe forum for people of almost all ages and health conditions to discuss their values regarding life, death, dying and what matters most.

## Highlights

• Gamification in advance care planning.
• *Hey du* is a low-threshold way to introduce advance care planning.
• Empowering people of almost all ages and health conditions to engage with ACP.
• *Hey du* can help overcome the barriers often associated with advance care planning.

## 1 Introduction

Many people find it difficult to talk about the end of their own lives and their own wishes and preferences for medical and end-of-life care. Addressing the end of life with family and friends is often perceived as challenging [1,2], for example because of generational differences, different beliefs, and cultural taboos [3]. The aim of advance care planning (ACP) is to overcome this barrier by empowering and motivating people to address their individual wishes and preferences for their own medical and nursing care in the event that they are unable to give consent. These wishes and preferences should be discussed with loved ones and/or service providers and ideally documented in writing [4].

Knowing and communicating one's wishes and preferences for medical and nursing care is important for people of all ages and health conditions. While it may be difficult for young and healthy people (YHP) to imagine themselves in a serious health situation, it is important to be able to take into account the wishes and preferences that exist at that moment [5]. For older and already (chronically) ill people, there are a number of effective ACP interventions to guide them through the empowerment process: for example, guided conversations such as Respecting Choices ® [6] and ‘Let me decide’ [7]. For ACP for YHP, however, there is little evidence or research to date, although it is recommended to go through the ACP process before the onset of a serious illness and to be prepared in the event of an acute emergency [2]. A key part of the ACP approach is that the process of considering and expressing wishes and preferences can and should be iterative, because wishes and preferences can evolve over time and with changing circumstances [4].

Even if planning for their own medical and long-term care before death might be less urgent for YHP at first sight, increasing their awareness of ACP can have a positive impact in different ways: Engaging in ACP as a young adult could improve their own ACP process as they age. In addition, many people accompany family members, for example, as they age and at some point take on the role of caregiver. In this role, younger people can play an important part in initiating and facilitating ACP discussions with the person concerned [5]. It is important to initiate and conduct ACP discussions in order to learn about the wishes and preferences of the person concerned. Without prior discussion of wishes and preferences, providing information about the person's wishes can create a great deal of uncertainty. This uncertainty is often accompanied by psychological distress for the people involved [8], who are often relatives or others close to the person [9]. To go through an ACP process for oneself or to support others in doing so, there must be a willingness to engage with the topic.

Studies have shown that gamification is an effective approach to getting people of all ages to engage with emotionally difficult topics [10,11]. In the international context, a systematic review by Liu, Zhao, Yang, Chan [12] shows that gamification can be an effective way to support and initiate ACP conversations due to the safe forum that the game provides for such sensitive discussions [12]. One of the games included in the systematic review [12] is *Hello*. *Hello* is a conversation game in which the players are encouraged to talk about their values in life, death, dying, and what matters most to them [13]. The game is currently used primarily in the US, although it has been translated into Spanish, Czech, Thai, and Korean. Studies have shown that *Hello* is an effective, inexpensive, and safe approach to effectively engage people in conversations and discussions about the topics addressed in the game [[13], [14], [15]].

To our knowledge, there are no studies in Germany that test the gamification approach for ACP and/or aim to motivate and empower YHP to engage in ACP. To date, ACP services in Germany have mainly focused on the elderly or sick (e.g. Patientenverfügungsgesetz [16], the Hospice and Palliative Care Act [17], *Behandlung im Voraus planen* [18], the STADPLAN project [19]).

The aim of this study was twofold: The first aim was to translate the conversation game *Hello* to German, adapt it if necessary to the German context, and test its acceptability and comprehensibility via cognitive pretesting. Secondly, two observational studies were conducted to gain further insight into the acceptability and comprehensibility of *Hey du,* the German version of *Hello*. Considering the important role that culture plays in communication about end-of-life issues, it is important to consider potential differences between nationalities [20] [21]. Furthermore, this study was designed to evaluate whether adaptations are needed to adapt the game to the German context in addition to the language translation.

## 2 Methods

The methodological approach of this study is divided into three steps:

1) The conservation game *Hey Du* was systematically translated into German (see chapter 2.2).
2) An initial assessment of the acceptability and comprehensibility of the translation was carried out in a cognitive pre-test (see chapter 2.3).
3) Two observational studies were conducted to gain further insight into acceptability and comprehensibility (see chapter 2.4).

### 2.1 Description of the conversation game

The conversation game *Hey du* is the German version of the commercially available English version of the conversation game *Hello* [22]. In the game, players answer 32 questions about their wishes and preferences regarding dying and end-of-life care (see Table 1). First, they answer these questions in writing for themselves and then discuss them in the group (2–5 people). Players can award ‘Thank You’ chips for responses they find thought-provoking, heart-warming, or otherwise worthy of expressing gratitude for sharing.

**Table 1:** Example questions from *Hey du.*

Question Number	Question
No. 4	Name the three-person committee who should be consulted on any decisions made about whether to continue life-saving care if you can't communicate?
No. 14	When you think about care at the end of your life, what do you worry more about: • Not getting enough care • Getting overly aggressive care • Other:
No. 21	What music do you want to be listening to on your last day alive?
No. 27	I want my doctor to be focused on maximizing: • The length of your life • The quality of your life • Other:

### 2.2 Translation process

For the translation of the game *Hey du*, the Translation, Review, Adjudication, Pretest, Documented (TRAPD) process was used [23], to ensure a structured and transparent translation process which could be replicated. In the translation phase, three groups (with two members per group) produced independent translations. These translations were then rated by three members of the research team during the review phase and merged into a single translation. In the adjudication phase, the translation was compared to the original. This was followed by a cognitive pretest. The entire process was documented.

### 2.3 Cognitive pretest

#### 2.3.1 Aim of the cognitive pretest

The aim of the cognitive pretest was to obtain an initial assessment of the acceptability and comprehensibility of the translated conversation game *Hey du* and to explore potential adaptations needed in addition to language translation. The cognitive pretest included the think-aloud technique, probing techniques and paraphrasing to learn more about the cognitive response process of users [24]. To conceptualize acceptability, we used the theoretical framework of acceptability (TFA) [25], which defines acceptability as a multi-faceted construct that reflects the extent to with people assess an intervention to be appropriate.

#### 2.3.2 Recruitment

A convenience sample of individuals aged 18 and older was invited to participate in the cognitive pretest. Participants were recruited from the interviewer's personal network. The interviewers were motivated to approach people from their own age group as well as from their parents' generation in order to obtain a sample that was as age-diverse as possible. The participants first received information about the study in a personal conversation. If they were interested in participating, they then received more information about the study and a consent form in writing. The consent form was signed by the participants before the study was conducted.

#### 2.3.3 Data collection

For the cognitive pretest, *n* = 12 cognitive interviews were conducted. The interviews were conducted by trained graduate students under the supervision of the research team (JJ, JKN). During the cognitive pretest, participants were presented with each of the game questions one at a time. Participants were asked to use the think-aloud technique and to explain how they understood the question they had just been asked and what information they thought the question was trying to elicit. Questions that were considered potentially difficult to understand should also be paraphrased. Participants were further asked to reflect on the acceptability of the questions and the gaming process as a whole. Age, gender, and previous experience with ACP were also collected. At the end of the pretest, participants were given the opportunity to review all questions and the game instructions again and make suggestions for changes.

#### 2.3.4 Data analysis

The pretests were transcribed using Kuckartz's [26] methods of transcription and then analysed qualitatively. The transcripts were analysed using directed content analysis [27] using Excel 2021 to identify problems in understanding the questions and the need for changes.

### 2.4 Observational studies

#### 2.4.1 Aim of the observational studies

The aim of the observational studies was to gain further insight into the acceptability using three component constructs of the TFA: (1) perceived effectiveness, defined as the extent to which *Hey du* is considered likely to achieve its intended purpose; (2) affective attitude, defined as a person's feeling about the intervention; and (3) self-efficacy, which is a person's confidence that he or she can perform the expected actions [25]. Furthermore we explore the comprehensibility of *Hey du*.

#### 2.4.2 Study design

We evaluated the acceptability and comprehensibility of *Hey du* during two observational studies, involving populations that tend to be younger and healthier than the current main target groups of evaluated ACP interventions. The first study was conducted with nursing students to test the acceptability and comprehensibility of using *Hey du* in healthcare education. It is possible that *Hey du* not only changes personal ACP behaviour, but also has a positive effect on the preparation and conduct of ACP discussions by healthcare professionals. The second study was conducted in a family/friendship context.

#### 2.4.3 Recruitment

Study 1: A nursing school that had already shown interest in the *Hey du* conversation game was invited to participate in the study. The contact person for the study team at the school selected a class of nursing students whose curriculum would be a good thematic fit for the conversation game according to the nursing school. Students in the selected class were informed the week before the game event that their class would be participating in a game event during class time for approximately three hours. They were introduced to the game and the scientific monitoring for the game event. After the short presentation, the students were given the written study information and consent forms. Participation in the game *Hey du* was mandatory as part of the teaching curriculum, but the students were given the option to participate in the scientific survey or not. All students agreed to take part in the scientific survey. The signed consent forms were collected at the game event.

Study 2: A convenience sample was recruited. The study team approached people in their personal environment (family members, friends and friends of friends who were interested in participation), and invited them to participate in the study. If they were interested, they were sent the study information and the informed consent form. The signed consent form was collected before the start of the game event.

#### 2.4.4 Outcome measure

A paper-based questionnaire was used to assess comprehensibility and acceptability. Participants were asked to indicate, on a 4-point scale (*strongly agree* to *strongly disagree*), the extent to which they agreed with statements about the *Hey du* conversation game.

##### 2.4.4.1 Comprehensibility

Comprehensibility was measured by assessing the extent to which participants agreed that the questions asked in the game were understandable to them. In a free text field, participants could indicate which questions they thought were hard to understand or not understandable at all.

##### 2.4.4.2 Acceptability

When asked about the acceptability of the game, participants indicated the extent to which they agreed with statements about the following four aspects:

1. *Appropriateness for discussing wishes and preferences regarding medical and nursing care at end of life* (TFA: perceived effectiveness). Participants indicated the extent to which they agreed with the statement that the game was appropriate for discussing a sensitive topic such as dealing with one's own wishes and preferences regarding medical and nursing care at the end of life.
2. *Helpfulness in understanding one's own wishes and preferences regarding medical and nursing care at end of life* (TFA: perceived effectiveness). Participants indicated the extent to which they agreed with the statement that the game helped them come to terms with their wishes and preferences regarding their own future medical and nursing care.
3. *Comfort while playing* (TFA: affective attitude). Participants indicated the extent to which they agreed with the statement that they felt comfortable while playing *Hey du*.
4. *Potential use of the game in families or with friends or initially with strangers* (TFA: self-efficacy). Participants indicated the extent to which they agreed with the statement that they could imagine playing the game (1) with family members, friends, or other close people; or (2) with people who were strangers at the beginning of the game.

#### 2.4.5 Data collection

Study 1: At the beginning of the game event, the game and the study procedure was explained to the students in detail. The game was played in two groups of five and one group of four. After the game, the students (*n* = 14) were given a short survey about the comprehensibility and acceptability of the game. In addition, there was a semi-structured feedback session for which field notes were prepared. In the feedback session, participants were asked to provide feedback on the following topics: (1) general game experience, (2) use of the game in the family environment and/or with strangers, (3) impact of *Hey du* on their own ACP behaviour, and (4) use of *Hey du* in their future work as nurses. Eight weeks after the game, they were given another short survey by the contact person at the nursing school. In this survey, they were asked whether they found *Hey du* helpful in understanding their own wishes and preferences for medical and nursing care in the event of incapacity and whether they thought *Hey du* was an appropriate format for this sensitive topic.

Study 2: Study 2 used a convenience sample (*n* = 50). Family members and groups of friends were invited to play *Hey du*. The games took place in the participants' private spaces. As in study 1, participants were surveyed immediately after completing the game and again four weeks later.

#### 2.4.6 Data analysis

Frequencies and mean scores of the data were calculated for comprehensibility and acceptability with STATA SE15. Missing data were not imputed. The field notes of the feedback session from study 1 were analysed using directed content analysis [27] with regard to the topics discussed in the feedback session: (1) general game experience, (2) use of the game in the family environment and/or with strangers, (3) impact of *Hey du* on participant's own ACP behaviour, and (4) use of *Hey du* in their future work as nurses.

### 2.5 Ethical approval

Ethical approval was obtained from the ethics committee of the University of Wuppertal (cognitive pretest: SK/AE 230125 [15th February 2023]; study 1: SK/AE 230809 [8th November 2023]; study 2: SK/AE 230725 [14th August 2023]). All methods were performed in accordance with these approvals. Informed content was obtained from all participants.

## 3 Results

### 3.1 Cognitive pretest

#### 3.1.1 Study population

The cognitive pretest was conducted with 12 people. Seven of the participants were female (58.3 %), five were male (41.7 %). The mean age was 37 years (*SD* = 17.49). Fifty percent of the participants had already completed ACP documents.

#### 3.1.2 Results of the cognitive pretest interviews

The participants had no major problems understanding the questions. They were able to reproduce the questions in their own words and could imagine the content issues they would address when answering the questions.

Three of the 32 questions required minor changes. One question was made easier to read by inserting an extra line between two sentences, another question had a word replaced to make the question clearer, and the third question was slightly shortened. Cultural adaptations were not necessary. The questions were rated as understandable by the participants.

### 3.2 Observational studies

#### 3.2.1 Study population

Table 2 shows the descriptive characteristics of the study population. More than half (57.6 %) of participants were female. One third (36.4 %) was between 18 and 27 years of age. In study 1, the proportion of 18–27-year-olds (62.5 %) was significantly higher than in study 2.

**Table 2:** Descriptive characteristics of the study population of the observational studies.

	Total *n* = 66 (%)	Observational study 1 *n* = 16 (%)	Observational study 2 *n* = 50 (%)
Age group			
18–27	24 (36.4)	10 (62.5)	14 (28.0)
28–37	14 (21.2)	1 (6.3)	0 (0.0)
38–47	1 (1.5)	1 (6.39	13 (26.0)
48–57	9 (13.6)	0 (0.0)	9 (18.0)
58–67	14 (21.2)	0 (0.0)	14 (28.0)
N/A	4 (6.1)	4 (25)	0 (0.0)
Gender			
Woman	38 (57.6)	10 (62.5)	28 (56.0)
Man	24 (36.4)	2 (12.5)	22 (44.0)
N/A	4 (6.1)	4 (25)	0 (0.0)

#### 3.2.2 Comprehensibility

Nearly all of the participants (98.5 %, *n* = 65) strongly or somewhat agreed with the statement that the questions asked in the game are understandable. Only one participant (1.5 %) indicated that he or she tended to disagree with this statement. Two people said they were not sure what the term ‘non-medical facts’ meant, which appears in one of the 32 questions without further explanation. One participant stated that some questions covered similar content.

#### 3.2.3 Acceptability

**Fig. 1:**
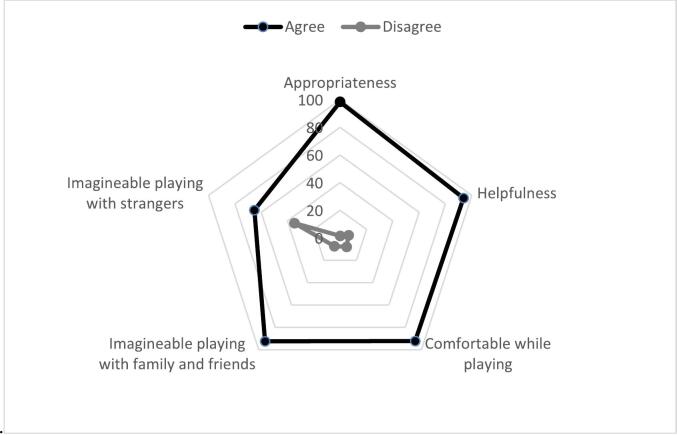
Acceptability of *Hey du.*

Fig. 1 shows, that nearly all of the participants (95.4 %, *n* = 62) strongly or somewhat agreed with the statement that *Hey Du* is suitable for discussing a sensitive topic such as dealing with one's own wishes and preferences regarding medical and nursing care at the end of life. One participant (1.6 %) stated that he did not tend to agree with this statement. Most of the participants (93.7 %, *n* = 59) found the game helpful in understanding their wishes and preferences for future medical and long-term care. In each study, two participants (3.2 %) found the game to be not entirely helpful or not helpful at all. Moreover, 92.1 % of participants (*n* = 58) stated that they fully or somewhat agreed with the statement that they felt comfortable while playing; 7.9 % (*n* = 5) tended to disagree with this statement. In addition, 92.4 % (*n* = 61) of participants strongly or somewhat agreed that they could imagine playing the game with family members, close friends, or other people close to them; three people (4.6 %) tended to disagree with this statement, and two (3.0 %) strongly disagreed. Nearly two-thirds of the participants (65.2 %, *n* = 43) agreed with the statement that they could imagine playing the game with people who were strangers to them at the beginning of the game; 24.2 % (*n* = 16) stated that they disagreed with this statement and 10.6 % (*n* = 7) that they strongly disagreed with this statement.

Four topics were discussed during the feedback session. Topic 1 was general feedback on the game experience. The atmosphere of the game was described as relaxed. It was regarded as positive that a question is answered first for oneself before it is shared with others on a voluntary basis. The second topic was the use of the game in the family and with other close people. It was mentioned that the game might be a good starting point for conversations with one's own family to learn more about people's wishes and preferences. The third topic was the benefits of *Hey du* for one's ACP. It was mentioned that the game had given them ideas to think more deeply about in regard to their own end of life, death, and the wishes and preferences associated with it. It was also mentioned that the first thoughts about ACP had already been formulated in writing through the game and that the barrier to communicating this to a doctor had now been lowered. The fourth topic discussed by the nursing student participants was whether and how *Hey du* could be used with patients to facilitate access to patients for discussions about ACP. The nursing students suggested that *Hey du* would help them approach the topic of ACP with patients and saw the game as an opportunity to get to know patients better as people. They mentioned the possibility that patients could first play the game alone or with their families and then talk to the nurse about the game.

## 4 Discussion and conclusion

### 4.1 Discussion

The results of the cognitive pretest and the two observational studies show that the conversation game *Hey du* is comprehensible and acceptable. The vast majority of participants (>92 %) in the studies stated that *Hey du* helped them to come to terms with their own wishes and preferences for medical and nursing care and that they felt comfortable playing. These results are consistent with the US studies that tested the feasibility and acceptability of the conversations using an English-version of *Hello* [13,15,28].

The results of our study do not replace an effectiveness evaluation of the game. Nevertheless, they show that, from the participants' point of view, there are no reservations about using *Hey du* with a group of people who are not in need of care and who are not seriously ill. Only a few adaptations were necessary to make *Hey Du* comprehensible and acceptable in the German context. The results also show that the time frame necessary to play *Hey du* and to engage with the topics of ACP and end-of-life issues is appropriate, even if it is a topic that is often associated with discomfort [29]. While *Hey du* is not a holistic ACP intervention, these results suggest that it may be an appropriate tool to empower and motivate people to engage with ACP, to start reflecting and formulating their own wishes and preferences, and to learn to communicate them. This can help overcome social and cultural barriers that otherwise exist in relation to ACP [3]. *Hey du* could be a way to reach people of all ages, almost regardless of their state of health, especially people who have not yet had to deal with the topic, such as YHP. By making the game available to YHP in a systematic way it could increase their engagement and awareness of ACP and, as a result, strengthen ACP planning for their own future. As a result of the increased awareness of ACP and the empowerment of YHP, it is conceivable that the game will also lead to conversations with elderly and/or sick family members and relatives being initiated and facilitated. Possible implementation strategies should be explored in further studies.

The results of the two studies show that the use of *Hey du* is not limited to one setting or to groups of people who know each other very well and have a close relationship. *Hey du* was well received in both the family and nursing school settings. This finding is also consistent with US studies in which the original game has been successfully used in different settings and contexts (e.g. [14,28,30]). A study by Van Scoy et al. in 2016, however, found mixed results when participants were asked with whom they could imagine playing *Hello*. Participants in game groups that initially consisted of strangers were more likely to say that they could not imagine playing the game with strangers. Participants who played *Hello* with people they knew were more likely to say they would not want to play the game with strangers [13]. In our study, more than half of the participants (65.2 %) indicated that they could imagine playing the game with people who were initially strangers to them.

The results of the pretest and the two observational studies suggest that *Hey du*, like *Hello* in the US [13,14], is an acceptable way for people to engage with ACP.

### 4.2 Innovation

To our knowledge, *Hey du* is the first scientifically supported, low-threshold gamification approach in Germany to introduce ACP to people of almost all ages and health conditions. Previous ACP programmes have mainly targeted older and/or (chronically) ill people [31]. The extension of the target groups is in accordance with the ACP concept, as it aims to empower and motivate people of all ages and health conditions to deal with their wishes and preferences regarding death and dying [4].

*Hey du* also motivates and empowers younger and healthy individuals to engage with ACP. This can improve their own ACP process as they get older and help them to start, lead, and facilitate ACP conversations with family members or close friends in the future. This can help to reduce the uncertainty associated with making medical and care decisions for another person and thus decrease the psychological stress for the person making the decision [8], who are often relatives or others close to the person [9].

### 4.3 Limitations

This study does not provide any insight into the effectiveness of *Hey Du* as it is not a randomised controlled trial (RCT). This would require further, larger studies to be conducted in the future. A more detailed description of the samples demographic is not possible due to the small size in order to ensure data protection.

The generalizability of the results is limited, because participants were selected for the cognitive pretest and the observational studies using convenience sampling. In the cognitive pre-test sample, 50 % of the participants had already completed ACP documents. It is possible that these people found it easier to understand the questions and rate *Hey du* as acceptable than people who had not completed ACP documents. Something similar may be true for sample 1. Due to the professional background of the students, the assessment of comprehensibility and acceptability could be biased.

In addition, only sample 1 could be offered a structured feedback session, because sample 2 played in small groups. In order to schedule these, a feedback session had to be omitted.

Nevertheless, based on the results of the cognitive pretest and the observational studies together with the study results on the original *Hello* game from the US [13,14], it can be concluded that the use of *Hey du* to initiate ACP conversations is acceptable and comprehensible.

### 4.4 Conclusion

This study shows that *Hey du* is an acceptable and comprehensible approach to help individuals engage in ACP and end-of-life discussions, and thus the gamification approach seems to work with ACP in Germany. To be able to make a statement about the effectiveness of the game, however, a randomised controlled trial needs to be conducted. A RCT could include the game either as an intervention or as an implementation strategy in a trial testing another ACP intervention.

## CRediT authorship contribution statement

**Julia Jaschke:** Writing – original draft, Project administration, Methodology, Investigation, Formal analysis, Conceptualization. **Sara Söling:** Writing – review & editing, Methodology. **Juliane Köberlein-Neu:** Writing – review & editing, Supervision, Methodology, Conceptualization.

## Declaration of generative AI and AI-assisted technologies in the writing process

During the preparation of this work, the authors used DeepL to improve the linguistic quality of the manuscript before the proofreading. After using this tool, the authors reviewed and edited the content as needed and take full responsibility for the content of the publication.

## Funding sources

This research did not receive any specific grant from funding agencies in the public, commercial, or not-for-profit sectors.

## Declaration of competing interest

The authors declare that they have no known competing financial interests or personal relationships that could have appeared to influence the work reported in this paper.

## Data Availability

The datasets used and/or analysed during the current study are available from the corresponding author on reasonable request.
